# Retrospective Investigation of Difficult Airway Cases Encountered in Bursa Uludag˘ University Medical Faculty Operating Room

**DOI:** 10.5152/TJAR.2023.22213

**Published:** 2023-04-01

**Authors:** Didem Alemdar, Selcan Akesen, Hülya Bilgin

**Affiliations:** 1 Clinic of Anaesthesiology and Reanimation, Bursa City Hospital, Bursa, Turkey; 2 Department of Anaesthesiology and Reanimation, Bursa Uludag˘ University School of Medicine, Bursa, Turkey

**Keywords:** Difficult airway, laryngoscopy, preoperative tests

## Abstract

**Objective:**

Ensuring airway patency and proper management of ventilation by anticipating difficulties that can occur in airway control are vital in prevent­ing anaesthesia-related complications. We aimed to determine the role of preoperative assessment findings in difficult airway management.

**Methods:**

In this study, critical incident records of difficult airway patients between 2010 and 2020 in the operat­ing room of Bursa Uludag˘ University Medical Faculty were retrospectively analysed. A total of 613 patients, whose records were fully accessible, were grouped as paediatric (under 18 years old) and adult (18 years and over).

**Results:**

The success rate for maintaining an airway in all patients was 98.7%. Pathological situations which cause difficult airways were head and neck region malignancies in adult patients and congenital syndromes in pae­diatric patients. Anatomical reasons that cause difficult airway were anterior larynx (31.1%) and short muscular neck (29.7%) in adult patients and small chin (38.0%) in paediatric patients. A significant statistical relationship was found between difficult mask ventilation and increased body mass index, male gender, modified Mallampati class 3-4, and thyromental distance <6 cm (*P* = .001, *P* < .001, *P* < .001, and *P* < .001, respec­tively). The correlation of Cormack–Lehane grading with modified Mallampati classification, upper lip bite test, and mouth opening distance was statistically significant (*P* < .001, *P* < .001, and *P* < .001, respectively).

**Conclusion:**

In male patients with increased body mass index, modified Mallampati test class of 3-4 and thyromental distance of <6 cm should suggest the possibility of difficult mask ventilation. In modified Mallampati classification and upper lip bite tests, the possibility of difficult laryngoscopy should be considered as class increases and mouth opening distance becomes shorter. Preoperative assessment, including a good history taken from the patient and a complete physical examination, is crucial to provide solutions for difficult airway management.

**Main Points**

- Establishing airway patency by anticipating the difficulties that may occur in airway control, and proper management of ventilation are vital in preventing anesthesia-related complications.
- Bedside measurements and evaluations in the preoperative period are valuable in predicting difficult airway.
- In male patients with an increased body mass index, the Modified Mallampati test class 3-4 and the thyromental distance <6 cm should suggest the possibility of difficult mask ventilation.
- As the modified Mallampati test and upper lip bite test class increase, the possibility of difficult laryngoscopy should be considered as the mouth opening distance becomes shorter.

## Introduction

In the UK, 16 airway-related deaths were reported during approximately 2.9 million general anaesthesia applications in 1 year (2008-2009), and accordingly, death probability was given as 1 in 180 000 general anaesthesia procedures.^1,2^

Up to the present, various methods have been developed, and algorithms have been created and updated in order to reduce mortality and morbidity related to airway management in patients.^3-5^

In this study, we tried to determine the demographic data, preoperative examination findings, results of bedside tests, and materials and techniques preferred for difficult airway management of difficult airway cases encountered in the operating room of Bursa Uludag˘ University Medical Faculty between August 2010 and July 2020. Based on the data obtained, we aimed to identify characteristics and findings that may assist us in the future.

## Methods

Following the approval of Bursa Uludag˘ University Non-Interventional Clinical Research Ethics Committee dated September 30, 2020, and numbered 2020-17/21, the digital files of difficult airway cases encountered in the operating room of Bursa Uludag˘ University Medical Faculty Hospital between August 2010 and July 2020 were reviewed retrospectively. The demographic information of the patients and their experiences during airway management were obtained by examining the critical incident from Bursa Uludag˘ University Anaesthesiology and Reanimation Department. Our study was retrospective, and informed consent was not obtained from the patients.

The files of 710 patients whose difficult airway information was found to have been completed on the critical incident form were examined, and 613 patients whose files could be accessed were included in the study.

### Statistical Analysis

All obtained data were recorded in the Statistical Package for the Social Sciences program version 23.0 file (IBM SPSS Corp., Armonk, NY, USA). Demographic and categorical data are given as frequency (n) and percentage (%). The Chi-square test was used for the correlation analysis of the categorical data and Spearman correlation analysis was used for the correlation analysis of the numerical data.

## Results

Of the 613 patients whose records were examined, 63.1% were men. The mean age of the patients was 47.3 years (male: 47.6 years, female: 46.8 years). The mean ages of paediatric and adult patients were 5.6 years and 52.8 years, respectively.

When adult patients were examined, the rate of patients with body mass index (BMI) <18 was 6.2%, the rate of patients with BMI 18.5-24.9 was 31%, the rate of patients with BMI 24.9-29.9% was 30.1%, the rate of patients with BMI 30-34.9 was 19.4%, the rate of patients with BMI 35-39.9% was 8.5% and the rate of patients with BMI ≥40 was 4.8%.

When the patient records were analysed by excluding 15 paediatric and 271 adult patients without any systemic disease or pathology that could be associated with difficult airway, it was found that the highest rates associated with difficult airway were head and neck region malignancies (62.8%) in adult patients and congenital syndromes (51.8%) in paediatric patients (Table 1).

According to the records, unexpected difficult airway was detected in 23.9% of paediatric patients and 24.9% of adult patients. When the bases of expectation were examined in 461 patients with expectation of difficult airway, it was understood that 7.4% of all patients were found to be diagnosed by past history alone, 54.6% by physical examination, and 37.8% by physical examination and a past history together.

When the distribution of patients with difficult airway was examined according to surgical clinics, it was seen that adults underwent surgery mostly in otorhinolaryngology (27.6%), general surgery (19.9%), and plastic and reconstructive surgery (16.1%) clinics, while paediatric patients underwent surgery mostly in paediatric surgery (18.3%), otorhinolaryngology (15.5%), and cardiovascular surgery (14.1%) clinics.

It was determined that thyromental distance and mouth opening distance were measured and that the upper lip bite test was assessed in all adult patients (Table 2). It was observed that modified Mallampati assessment could not be performed due to limited mouth opening in 34 (6.3%) patients.

The distribution of anatomical and other features affecting anatomy that may cause difficult airway in all patients according to age groups was examined. The most common anatomical features causing airway difficulty were anterior larynx (31.1%), short muscular neck (29.7%), and reduced neck movement (29.7%) in adult patients and anterior larynx (35.0%) and small chin in paediatric patients (Table 3).

The rate of adult patients with difficult mask ventilation was 51% and the rate of paediatric patients was 33.8%. There was no record of mask ventilation in 8 of the patients who underwent awake intubation.

It was seen that in general, at the end of all attempts, the success rate of establishing an airway was 98.7% and that the success rate at the first attempt was generally 36.9% (26% in paediatric patients and 36.5% in adult patients).

When the success rates of instruments used for endotracheal intubation in patients with a successful airway were examined, these were found to be 36.3% with conventional laryngoscopy, 84% with video laryngoscope, and 88.3% with fibreoptic bronchoscope. The operations of 8 patients (2 paediatric patients and 6 adult patients) with failed airway were postponed.

General anaesthesia was administered to 75.8% of adult patients and 92.9% of paediatric patients during airway management, while 24.2% of adult patients were awake (with sedation and local anaesthesia). Airway control was achieved while awake in 5 of the paediatric patients, with a mean age of 13.6 years.

Cormack–Lehane (CL) class was found to be 1 in 1.8%, 2 in 6.6%, 3 in 25.7%, and 4 in 65.9% of 405 patients whose laryngoscopic appearances were evaluated. It was determined that CL classification could not be assessed in 133 patients with an expectation of a difficult airway and who planned to undergo awake FOBI, 2 patients in whom a supraglottic airway was used, and 2 patients who underwent surgical tracheotomy with mask ventilation support.

It was determined that among the patients who were successfully intubated, 510 (53 paediatric and 457 adult patients) were extubated without any problems and that there were 49 patients (13 paediatric and 36 adult patients) who were admitted intubated to the intensive care unit and 46 patients (3 paediatric, 43 adult patients) who underwent tracheotomy during the operation due to their existing pathologies. It was found that among the patients who were taken intubated to the intensive care unit, 7 patients (4 adult patients and 3 patients under 18 years of age) were taken for close follow-up due to difficult airway, while the other patients were those who were evaluated together with the surgical team and for whom it was decided to transfer them to the intensive care unit as intubated due to long-term surgeries planned with controlled extubation such as coronary artery bypass graft surgery and intracranial mass surgery.

The relationships of BMI values with preoperative screening tests and CL classification of laryngoscopic views were investigated in adult patients included in the study. The relationship between BMI and modified Mallampati classification (*P* = .001), thyromental distance measurements (*P* < .001), upper lip bite test (*P* < .001), and CL classification was found to be statistically significant (*P* < .001) (Table 4).

In adult patients, the relationship of face mask ventilation with demographic data (BMI and gender), preoperative screening tests, and CL classification was investigated. In the adult patients screened in the study, the relationship between face mask ventilation and BMI (*P* = .001), gender (*P* < .001), modified Mallampati classification values (*P* < .001), and thyromental distance values (*P* < .001) was found to be statistically significant (Table 5).

In adult patients, the relationship of preoperative screening tests with CL classification (394 enrolled adult patients) was examined. The correlation of modified Mallampati classification, upper lip bite test, and mouth opening distance with CL classification was found to be statistically significant (*P* < .001, *P* < .001, and *P* < .001, respectively) (Table 6).

## Discussion

Failures in airway management during general anaesthesia applications are the most important cause of anaesthesia-related complications in patients.

Past history and physical examination findings that can predict the presence of a difficult airway in patients prior to anaesthesia applications are very important. In the presence of a difficult airway, the routes to be followed and the additional methods and materials to be applied must be carefully planned. For this, a well-equipped operating room and an experienced clinician are required.

Of the 613 patients whose records were examined, 88.4% were in the adult group and 11.6% were in the paediatric group. The male sex ratio was found to be high in both age groups, and among all patients, the number of male patients was 387 (63.1%). In a review by Durbin et al.^6^ when more than 53 000 patients who underwent airway management were examined, it was revealed that male gender was significantly higher. In some studies, no relationship between gender and difficult airway could be found.^7,8^

When the BMI values of the adult patients whose records we scanned were examined, it was found that they were normal and/or overweight at a high rate (61.2%). Although it has been found in a number of studies^9,10^ that an increased BMI is an increased risk factor for difficult airway, the obesity rate among our patients was low. It was thought that one of the reasons for this might be additional systemic diseases or pathologies that were present and that could cause a difficult airway in most of the patients. When the patient records were examined in detail, it was observed that 50% of patients aged 18 and over had systemic diseases or pathologies that could cause a difficult airway, such as head and neck malignancy, obstructive sleep apnoea syndrome, or ankylosing spondylitis. However, only 11% of the unexpected difficult airway patients (most frequently obstructive sleep apnoea syndrome) had an additional systemic disease or pathology.

When patients with increased BMI were evaluated, modified Mallampati class, thyromental distance, and upper lip bite test values evaluated in the preoperative period were found to be significantly higher in this patient group. The reason for this may be the increase in soft tissue around the neck, the presence of a large tongue, a relatively small mandible, and a decrease in jaw subluxation due to adipose tissue in obese patients. In a study by De Jong et al.^11^ it was reported that the modified Mallampati class value was 3-4 in 84 (52%) of 160 patients with difficult intubation in obese patients who were operated on in the operating room.

In the study, it was found that in the adult patient group, the clinics with the highest number of difficult airway cases were otorhinolaryngology (27.6%), general surgery (19.9%), and plastic and reconstructive surgery (16.1%) clinics. This may be due to the fact that procedures such as airway interventions, head and neck malignancy surgeries, and thyroid gland surgeries are frequently performed in the relevant departments. Paediatric surgery (18.3%), otorhinolaryngology (15.5%), and cardiovascular surgery (14.1%) clinics were the most common clinics in paediatric patients. Since these are the clinics that perform the most frequent surgical interventions in patients in this age group, this was an expected result.

In the preoperative period, bedside measurements and assessments are also valuable in predicting difficult airway. In our clinic, some of these measurements (modified Mallampati classification, thyromental distance, mouth opening distance, and upper lip bite test) are made and recorded in adult patients in the outpatient clinic examination in elective surgeries and in the operating room in emergency cases. In the study, when the records were examined, it was determined that the rate of patients with modified Mallampati classification 3-4 (378 patients, 69.8%) was considerably high.

The importance of the modified Mallampati classification has been emphasised in studies on the preoperative determination of difficult airway. In a study conducted by Fritscherova et al.^8^ 67 patients (90.5%) with modified Mallampati classification 3-4 were reported in 74 difficult intubation cases. In a study carried out by Ramachandran and Kheterpal ^12^ it was reported that 308 (49%) out of 698 patients with both difficult mask ventilation and difficult intubation had modified Mallampati classification 3-4 and that this relationship was found to be statistically significant.

Again, when the thyromental distance, mouth opening distance, and upper lip bite test values measured in the preoperative period were examined, it was seen that contrary to expectations, in 64.8% of adult patients, thyromental distance was measured as ≥6 cm, in 68.6% of them, mouth opening distance was measured as ≥3 cm, while in 64.7% of them, the upper lip biting test was evaluated as class 1. This showed us that the causes of difficult airway in these patients were mostly concomitant systemic diseases or pathologies in 50% of the patients. In addition, it can be predicted that the people making the measurements and their evaluations may be different.

In a study made by Yıldız et al.^13^ it was stated that if the thyromental distance is between 6 and 6.5 cm, the likelihood of difficult intubation is high, while if it is below 6 cm, intubation is not possible. The threshold value for mouth opening distance was determined as 4.5 cm. In a study conducted by Selvi et al.^14^ it was reported that the specificity of thyromental distance measurement was 67.5% and that the sensitivity was 64.9%. Khan et al ^15^ stated that the upper lip bite test they described had the advantages of being quickly applicable and objective. In a study conducted by Bilgin and Ozyurt^16^ on 500 non-obstetric patients, it was found that the modified Mallampati test and thyromental distance assessment had a sensitivity of 43% and 35% and a specificity of 93% and 95%, respectively.

Cormack–Lehane classification, which is determined by evaluating the views of the laryngeal structures, vocal cords, and epiglottis during laryngoscopy imaging, has been used in many studies to define difficult laryngoscopy. In our study, it was determined that 91.6% of 405 patients who were found to have a CL classification had a CL value of 3 or 4. When Balakrishnan et al ^17^ compared the preoperative tests recommended for the prediction of difficult airway in 2004 cases with the CL classification, they revealed a rate of difficult intubation of 12.8%. In a study by Combes et al,^18^ the number of patients with CL 3 or 4 among 160 difficult intubation cases was reported as 145 (90%), and this rate was similar to that in our study. When the mouth opening distance, which shows the movement of the temporomandibular joint, is limited, this may obstruct the view of the larynx. Studies have shown that the risk of difficult laryngoscopy is also increased in patients with an increased BMI.^19^ In our study, the relationship between CL classification and mouth opening distance was found to be statistically significant, and CL classification values were revealed to be significantly higher in patients with increased BMI. Cattano et al ^20^ found in their study that the risk of difficult laryngoscopy increased in patients with a mouth opening distance of less than 3.5 cm and limited mandible protrusion.

In our study, 24 (33.8%) paediatric and 260 (48%) adults were found to have had difficulty in mask ventilation. There was no record related to mask ventilation in 8 of the awake (with local anaesthesia and sedation support) intubated patients. In patients with difficult mask ventilation, it was determined that 67.5% of adults and 75% of paediatric patients had 2-handed mask ventilation together with oral airway placement as a solution. In a study conducted by David et al.^21^ it was reported that there were 124 patients (8.9%) with difficult mask ventilation among 1399 patients and that the 2-handed mask ventilation technique or the use of an extraglottic device was required. Many factors contribute to difficult mask ventilation. These have been shown in many studies to be high BMI, male gender, high modified Mallampati value, advanced age, presence of a beard, and absence of teeth.^19-22^ In our study, the relationship between BMI and face mask ventilation was statistically significant in 542 adult patients, and the relationship between male gender and face mask ventilation was also statistically significant. The risk of difficult mask ventilation was also significantly higher in patients with an increased modified Mallampati class and a thyromental distance of less than 6 cm.

In obese patients, the increase in adipose tissue in the neck causes restriction in neck movements and makes face mask ventilation difficult. In a study by Langeron et al.^23^ a BMI of over 26 kg m^–2^ was stated as an independent risk factor for difficult mask ventilation, while an increased modified Mallampati class was stated as a potential risk factor for difficult face mask ventilation. Difficult intubation rates were also found to be high in patients with difficult mask ventilation.^23,24^ However, in our study, a significant relationship was not found between face mask ventilation and CL classification in adult patients.

The extubation stages of difficult airway patients are also an important stage of airway management and should be planned in advance. When examining the postoperative status of 613 patients whose records were examined, it was observed that 7 of the patients who were transferred to the intubated intensive care unit (49 patients, 8%) were transferred for close follow-up due to difficult airway and that the other patients were transferred due to reasons related to the operation, while a high percentage of patients (510 patients, 83.2%) were extubated without any problem.

In conclusion, in male patients with increased BMI, a modified Mallampati test class of 3-4 and a thyromental distance of <6 cm should suggest the possibility of difficult mask ventilation. As the modified Mallampati test class and upper lip bite test class increase, and the mouth opening distance becomes shorter, the possibility of difficult laryngoscopy should be considered. In terms of difficult airway, each patient should be carefully examined during the preoperative preparation period, and problems that may arise and possible ways to solve these should be planned. Considering that no single test is reliable on its own, patients should be examined as a whole, and it should not be forgotten that the use of difficult airway devices should be mastered in line with clinical facilities.

## Figures and Tables

**Table 1.** Distribution of Systemic Disease or Existing Pathologies That Could Cause Difficult Airway in Patients

Systemic Diseases or Pathologies	Paediatric		Adult		Total
	n	%	n	%	
Head and neck malignancy	2	3.6	166	62.8	168
Obstructive sleep apnoea syndrome	0	0	30	11.3	30
Ankylosing spondylitis	0	0	24	9.1	24
Cervical surgery and trauma	7	12.5	21	8.0	28
Arthritis	0	0	6	2.3	6
Acromegaly	0	0	5	1.9	5
Burns	3	5.3	4	1.5	7
Mediastinal mass	1	1.8	3	1.1	4
Infection in the head and neck area	0	0	1	0.4	1
Facial paralysis	0	0	1	0.4	1
Multinodular goitre	0	0	1	0.4	1
Congenital syndromes	29	51.8	1	0.4	30
Scoliosis	2	3.6	1	0.4	3
Cleft palate and lip	12	21.4	0	0	12
Total	56	100	264	100	312

**Table 2.** Distribution of Modified Mallampati Classification, Thyromental Distance and Mouth Opening Distance Measurements, and Upper Lip Bite Test Values in Adult Patients

		Frequency (n)	Percentage (%)
Modified Mallampati class	1	25	4.6
	2	105	19.3
	3	153	28.3
	4	225	41.5
	Unevaluable	34	6.3
Thyromental distance	≥6 cm	351	64.8
	<6 cm	191	35.2
Mouth opening distance	≥3 cm	372	68.6
	<3 cm	170	31.4
Upper lip bite test	1	351	64.7
	2	144	26.6
	3	47	8.7

**Table 3.** Distribution of Anatomical and Other Features Affecting Anatomy That Could Cause Difficult Airway According to Age Groups

Anatomical Features	Paediatric		Adult		Total
	n	%	n	%	
Anterior larynx	25	35	169	31.1	194
Reduced neck movement	16	22.5	161	29.7	177
Short muscular neck	1	1.4	161	29.7	162
Limited mouth opening	17	24	143	26.3	160
Immobile epiglottis	5	7	110	20.2	115
Small chin	27	38	74	13.6	101
Large tongue	5	7	66	12.1	71
Wide epiglottis	3	4.2	22	4.1	25
Protruding front teeth	9	12.6	26	4.7	35
Weak loose teeth	1	1.4	10	1.8	11
Absence of teeth	2	2.8	5	0.9	7
Prognathism	17	24	5	0.9	22
Other					
Radiotherapy history	0	0	56	10.3	56
Previous surgery	3	4.2	76	14.1	79
Previous tracheotomy	1	1.4	18	3.3	19

**Table 4.** Correlation Between Adult BMI and Modified Mallampati Classification, TMD Measurement, ULBT, and CL Classification Values

BMI		<18.5 (Underweight)	18.5-24.9 (Normal Weight)	25-29.9 (Overweight)	30-34.9 (Class 1 Obesity)	35-39.9 (Class 2 Obesity)	≥ 40 (Class 3 Obesity)	*P*
		n (%)	n (%)	n (%)	n (%)	n (%)	n (%)	
Modified Mallampati	Class 1	1 (4.0)	12 (48.0)	7 (28.0)	2 (8.0)	1 (4.0 )	2 (8.0)	.001
	Class 2	4 (3.8)	48 (45.7)	32 (30.5)	17 (16.2)	4 (3.8)	0 (0)	
	Class 3	6 (3.9)	32 (20.9)	55 (35.9)	38 (24.8)	15 (9.8)	7 (4.6)	
	Class 4	19 (5.9)	62 (30.4)	58 (30.0)	46 (20.1)	24 (8.7)	16 (4.9)	
TMD	<6 cm	9 (26.5)	43 (25.6)	55 (33.7)	46 (43.8)	17 (37.0)	21 (80.8)	<.001
	≥6 cm	25 (73.5)	125 (74.4)	108 (66.3)	59 (56.3)	29 (63.0)	5 (19.2)	
ULBT	Class 1	14 (41.2)	97 (57.7)	108 (66.3)	75 (71.4)	37 (80.4)	20 (76.9)	<.001
	Class 2	18 (52.9)	45 (26.8)	46 (28.2)	20 (19.0)	9 (19.6)	6 (23.1)	
	Class 3	2 (5.9)	26 (15.5)	9 (5.5)	10 (9.5)	0 (0)	0 (0)	
CL	Class 1	0 (0)	4 (57.1)	1 (14.3)	0 (0)	2 (28.6)	0 (0)	<.001
	Class 2	3 (11.1)	7 (25.9)	5 (18.5)	5 (18.5)	1 (3.7)	6 (22.2)	
	Class 3	3 (2.9)	26 (25.0)	35 (33.7)	22 (21.2)	12 (11.5)	6 (5.8)	
	Class 4	8 (3.0)	80 (30.0)	85 (31.8)	58 (21.7)	26 (9.7)	10 (3.7)	

BMI, body mass index; CL, Cormack–Lehane classification; TMD, thyromental distance; ULBT, upper lip bite test.

**Table 5.** Correlation Between Face Mask Ventilation and BMI, Gender, Modified Mallampati Classification Values, and TMD Values in Adult Patients

MV		Easy	Difficult	*P*
		n (%)	n (%)	
BMI	<18.5 (underweight)	14 (5.4)	19 (6.9)	.001
	18.5-24.9 (normal weight)	70 (26.9)	95 (34.7)	
	25-29.9 (overweight)	64 (24.6)	96 (35.0)	
	30-34.9 (class 1 obesity)	61 (23.5)	43 (15.7)	
	35-39.9 (class 2 obesity)	30 (11.5)	16 (5.8)	
	≥40 (class 3 obesity)	21 (8.1)	5 (1.8)	
Gender	Female	70 (26.9)	120 (43.8)	<.001
	Male	190 (73.1)	154 (56.2)	
Modified Mallampati	Class 1	9 (3.7)	15 (5.8)	<.001
	Class 2	29 (11.9)	75 (29.0)	
	Class 3	80 (32.9)	71 (27.4)	
	Class 4	125 (51.4)	98 (37.8)	
TMD	<6 cm	123 (47.3)	63 (23.0)	<.001
	≥6 cm	137 (52.7)	211 (77.0)	

BMI, body mass index; MV, mask ventilation; TMD, thyromental distance.

**Table 6.** Correlation Between CL Classification and Modified Mallampati Classification, ULBT, and MOD in Adult Patients

CL		Class 1	Class 2	Class 3	Class 4	*P*
		n (%)	n (%)	n (%)	n (%)	
Modified Mallampati	Class 1	2 (9.5)	2 (9.5)	7 (33.3)	10 (47.6)	<.001
	Class 2	1 (1.1)	7 (7.4)	23 (24.5)	63 (67.0)	
	Class 3	1 (0.8)	5 (3.9)	38 (29.4)	85 (65.9)	
	Class 4	3 (2.0)	10 (6.7)	36 (24.0)	101 (67.3)	
ULBT	Class 1	4 (1.3)	16 (5.2)	90 (29.1)	199 (64.4)	<.001
	Class 2	3 (3.8)	10 (12.6)	12 (15.2)	54 (68.4)	
	Class 3	0 (0)	1 (5.9)	2 (11.8)	14 (82.3)	
MOD	≥3cm	5 (1.3)	21 (5.6)	93 (25.0)	206 (55.4)	<.001
	<3cm	2 (1.2)	6 (3.5)	11 (6.5)	61 (35.9)	

CL, Cormack–Lehane classification; MOD, mouth opening distance; ULBT, upper lip bite test.

## References

[1] Cook TM Woodall N Frerk C , Fourth National Audit Project. Major complications of airway management in the UK: results of the Fourth National Audit Project of the Royal College of Anaesthetists and the Difficult Airway Society. Part 1: anaesthesia. Br J Anaesth. 2011;106(5):617 631. (10.1093/bja/aer058) 21447488

[2] Woodall NM Cook TM . National census of airway management techniques used for anaesthesia in the UK: first phase of the Fourth National Audit Project at the Royal College of Anaesthetists. Br J Anaesth. 2011;106(2):266 271. (10.1093/bja/aeq339) 21131655

[3] Apfelbaum JL Hagberg CA Caplan RA , et al. Practice guidelines for management of the difficult airway: an updated report by the American Society of Anesthesiologists Task Force on management of the difficult airway. Anesthesiology. 2013;118(2):251 270. (10.1097/ALN.0b013e31827773b2) 23364566

[4] Türk Anesteziyoloji ve Reanimasyon Dernegi (TARD). Anestezi uygulama Kılavuzları. Zor Hava Yolu. 2005;1:1 9.

[5] Frerk C Mitchell VS McNarry AF , et al. Difficult Airway ­Society 2015 guidelines for management of unanticipated difficult intubation in adults. Br J Anaesth. 2015;115(6):827 848. (10.1093/bja/aev371) 26556848 PMC4650961

[6] Durbin CG Bell CT Shilling AM . Elective intubation. Respir Care. 2014;59(6):825 46; discussion 847. (10.4187/respcare.02802) 24891194

[7] Aşık İ Göktuğ A Çanakçı N . Farklı entübasyon değerlendirme testlerinin zor entübasyon ile ilişkisi. Anest Derg. 2000;8(3):188 192.

[8] Fritscherova S Adamus M Dostalova K , et al. Can difficult ıntubatıon be easıly and rapıdly predıcted? Biomed Pap Med Fac Univ Palacky Olomouc Czech Repub. 2011;155(2):165 171. (10.5507/bp.2011.032) 21804626

[9] Riad W Vaez MN Raveendran R , et al. Neck circumference as a predictor of difficult intubation and difficult mask ventilation in morbidly obese patients: A prospective observational study. Eur J Anaesthesiol. 2016;33(4):244 249. (10.1097/EJA.0000000000000324) 26351829

[10] Lundstrøm LH . Detection of risk factors for difficult tracheal intubation. Dan Med J. 2012;59(4):B4431. 22459727

[11] De Jong A Molinari N Pouzeratte Y , et al. Difficult intubation in obese patients: incidence, risk factors, and complications in the operating theatre and in intensive care units. Br J Anaesth. 2015;114(2):297 306. (10.1093/bja/aeu373) 25431308

[12] Ramachandran SK Kheterpal S . Difficult mask ventilation: does it matter? Anaesthesia. 2011;66(suppl 2):40 44. (10.1111/j.1365-2044.2011.06933.x) 22074078

[13] Yıldız TŞ Çulha TH San S , et al. Zor entübasyonu belirlemede hangi testler daha güvenilirdir? Türk Anesth Rean Derg. 2006;34:162 168.

[14] Selvi O Kahraman T Senturk O . Evaluation of the reliability of preoperative descriptive airway assessment test in prediction of the Cormack-Lehane score: A prospective randomized clinical study. Jolurnal Clin Anesth. 2017;36:21 26. 10.1016/j.jclinane.2016.08.006 28183567

[15] Khan ZH Kashfı A Ebrahimkhani E . A Comparison of the upper lip bite test (a simple new technique) with modified Mallampati classification in predicting difficulty in endotracheal intubation: a prospective blinded study. Anesth Analg. 2003;96(2):595 599. (10.1097/00000539-200302000-00053) 12538218

[16] Bilgin H Ozyurt G . Screening tests for predicting difficult intubation. a clinical assessment in Turkish patients. Anaesth Intensive Care. 1998;26(4):382 386. (10.1177/0310057X9802600407) 9743852

[17] Balakrishnan KP Chockalingam PA . Ethnicity and upper airway measurements: a study in south Indian population. Indian J Anaesth. 2017;61(8):622 628. (10.4103/ija.IJA_247_17) 28890556 PMC5579851

[18] Combes X Jabre P Margenet A . Unanticipated difficult airway management in the prehospital emergency setting. Anesth. 2011;114:105 110. 10.1097/ALN.0b013e318201c42e 21169803

[19] El-Orbany M Woehlck HJ . Difficult mask ventilation. Anesth Analg. 2009;109(6):1870 1880. (10.1213/ANE.0b013e3181b5881c) 19923516

[20] Cattano D Killoran PV Cai C Katsiampoura AD Corso RM Hagberg CA . Difficult mask ventilation in general surgical population: observation of risk factors and predictors. F1000Res. 2014;3(204):204. (10.12688/f1000research.5131.1) 25485097 PMC4244761

[21] David ZF William HR Mary JJ , et al. Use of the intubating LMA-Fastrach 254 patients with difficult to manage airways. Anesthesiology. 2001;95:1175 1181. 11684987 10.1097/00000542-200111000-00022

[22] Sanuki T Watanabe T Ozaki Y , et al. Upside-down mask ventilation technique for a patient with a long and narrow mandible. Anesth Prog. 2014;61(4):169 170. (10.2344/0003-3006-61.4.169) 25517554 PMC4269358

[23] Langeron O Cuvillon P Ibanez-Esteve C Lenfant F Riou B Le Manach Y . Prediction of difficult tracheal intubation: time for a paradigm change. Anesthesiology. 2012;117(6):1223 1233. (10.1097/ALN.0b013e31827537cb) 23135259

[24] El Solh AA . Airway management in the obese patient. Clin Chest Med. 2009;30(3):555 568. (10.1016/j.ccm.2009.05.005) 19700052

